# Plant growth promoters mediated quality and yield attributes of milk thistle (*Silybum marianum* L.) ecotypes under salinity stress

**DOI:** 10.1038/s41598-021-02435-4

**Published:** 2021-12-01

**Authors:** Noreen Zahra, Abdul Wahid, Muhammad Bilal Hafeez, Mohammed Nasser Alyemeni, Tariq Shah, Parvaiz Ahmad

**Affiliations:** 1 Department of Botany, University of Agriculture, Faisalabad, 38040 Pakistan; 2 Department of Agronomy, University of Agriculture, Faisalabad, 38040 Pakistan; 3 College of Agronomy, Northwest A&F University, Yangling, 712100 China; 4 Botany and Microbiology Department, College of Science, King Saud University, P.O. Box 2460, Riyadh, 11451 Saudi Arabia; 5 Department of Agroecology, Universite de Bourgogne, 21000 Dijon, France; 6 Department of Botany, GDC, Pulwama, Srinagar India

**Keywords:** Plant stress responses, Salt

## Abstract

*Silybum marianum* (L.) Gaertn (Astraceae) is a well-reputed medicinal plant mostly utilized for silymarin (Sily) content and oil production, however, the information about Sily contents in achene part is still fragmented under different climatic conditions. In this study four milk thistle ecotypes from Faisalabad (FSD), Gujranwala (GUJ), Quetta (QTA), and Kallar kahar (KK) having an altered achene color were analyzed under salt stress*.* Application of plant growth promoters (PGPs) is one of the solution for ameliorating the effect of salinity and increasing the quantity and quality traits of milk thistle, so ascorbic acid (AsA), thiourea (TU), and moringa leaf extract (MLE) were soil supplied after developing salinity stress (120 mM with irrigation) at germination stage. Predetermined levels were selected for PGPs such as AsA (500 µM), MLE (3%), and TU (250 µM). Results revealed that all yield related attributes were significantly decreased, while secondary metabolites, pericarp epidermis, pericarp parenchyma, and pericarp seed integument increased under salinity stress. Data suggested that PGPs treatment was helpful to alleviate the deleterious effects of salinity stress and enhance the milk thistle quality and quantity parameters. The ecotypic variations with altered achene color patterns represent an advantage for QTA ecotypes for higher Sily extraction under salt stressed conditions.

## Introduction

Globally the interest in the use of medicinal plants is increasing, and the economic impact of serving nutraceuticals and pharmaceutical industries has grown in the last few years^1^. Milk thistle is well known as an annual or biennial medicinal herbaceous plant, presently widespread in European countries, Southern Australia, Western Asia, North Africa, North and South America^2,3^. The seeds of milk thistle contain a rich group of flavonoids (Flavo) (apigenin, chrysoeriol, dihydrokaempferol, eriodyctiol, kaempferol, naringin taxifolin, and quercetin), tocopherol, sterols, proteins, unsaturated fatty acids (i.e., oleic acid (30%), and linoleic acid (60%)) and sugars (i.e., arabinose, galactose, glucose, fructose, rhamnose, sucrose, and xylose)^4,5^. Nevertheless, the important biologically active substance of milk thistle seed is silymarin (Sily), that is 1.5 to 3.0% of dry matter with a complex mixture of flavonolignans (i.e., isosilybin A and B, silybin A and B, silychristin A and B, and silydianin)^6^. Milk thistle has been used as a medicinal plant as a remedy for various diseases such as anti-diabetic^7^, hepatoprotective^8^, hypocholesterolaemic^3^, anti-inflammatory^9^, and anti-cancer^10^.

Salinity stress is one of the most challenging scientific concerns that influence plant physiological and metabolical processes. It is expected that salinity covers about 50% of total agricultural land by 2050^11,12^. Salinity stress affects not only plant growth and development but also reduces grain yield and quality^13,14^. Plant under salinity reduces CO_2_ intake, photosynthesis, relative water, chlorophyll contents, which ultimately impairs cell division and elongation with the disturbance of the balance between antioxidants and reactive oxygen species that causes structural damages^15–18^. Under saline conditions, milk thistle (*Silybum marianum* (L.) reduced germination, root and shoot growth Hammami, et al.^19^. Ghavami and Ramin^20^ reported that 15 dS m^−1^ salinity decreased the achene weight and seed per capitula, total seeds plant^−1^, thousand achene weight, and total achene weight plant^−1^ in milk thistle. They also noted that the oil% was reduced while the Sily% and silybin% were increased. El-Garhy, et al.^21^ reported that salinity affects the branches numbers, capitulum number, and fruits dry weight in milk thistle. Gad and Abdelhaak^22^ observed the increase in inflorescences achene weight, total weight of achene/plant, silychristin, silydianin, silybin A and B, isosilybin A and B, and total Sily in milk thistle under salinity stress. Ghavami, et al.^23^ recorded that under saline conditions (12 dS m^−1^) the number of capitula/plant, main shoot capitulum's diameter, and seed yield/plant was decreased in milk thistle.

Several approaches are used for the improvement of salinity tolerances in crops, such as application of plant growth promoters (PGPs) (in soil, foliar, priming, coating, and pelleting with osmo-protectants, hormones, and plant extracts), natural and synthetic mulches, cultivation of salt-tolerant varieties, flooding, crop rotation, organic manuring, growth-promoting rhizobacteria, and fertilization^24–26^. The PGPs modulate plant responses to abiotic stresses and regulate their growth, developmental and physio-chemical properties^27^. Thiourea (TU) is a synthetic compound that contains nitrogen (36%) and sulfur (42%), which has gained attention as a PGP to improve the growth and development of plants under different stresses^25^. Yadav, et al.^28^ observed that TU application under salt stress improved the wheat and pearl millet plant height, relative water content, total chlorophyll, proline with better grain yield while decreased lipid peroxidation.

Ascorbic acid [vitamin C (AsA)] is a nonenzymatic antioxidant having significant potential as a PGP in detoxification of ROS with modulating many essential functions in plants under stressful conditions^29^. Moringa (*Moringa oleifera* Lam.) is known as a miracle tree that is exploited as a bio-stimulant. Its leaves are a good source of minerals, amino acids, antioxidants, fatty acids, and crude protein^30,31^. Application of moringa leaf extract (MLE) improves nutrient uptake, photosynthetic pigments, protein content, and grain yield of wheat under saline conditions^32^. This study was conducted to evaluate the effect of salt stress on milk thistle yield production and its quality traits especially concerned with Sily and its oil contents by using exogenous application of different PGPs. The special concern was given to the milk thistle ecotypes having better quality traits under saline stress conditions.

## Materials and methods

### Experimental details

Milk thistle ecotypes were collected from diverse ecozones of Pakistan viz*.*, Faisalabad (FSD), Gujranwala (GUJ), Kallar Khar (KK), and Quetta (QTA). The three ecological zones were generally recognized in this study, cold semi-arid (QTA), semi-arid (FSD and KK), and hot semi-arid (GUJ)^33–36^. Evaluation of ecotypes was based on growing milk thistle populations in a new environment with its own genotypic and ecotypic behavior, under saline stress. It is certified that the milk thistle ecotype from QTA was provided by Arid Zone Research Institute, Quetta. The ecotype from Guj was collected from Gujranwala was seed material from a progressive grower and maintainer of milk thistle plants in the crop fields. The ecotype from KK was collected growing wild near Rest House located on the road from Kallar Kahar to Khushab. The ecotype from Faisalabad was obtained from the Department of Botany in the Botanical Garden, where it was growing as the medicinal herb. All these ecotype collections were submitted as voucher specimens to the herbarium maintained at the Department of Botany. The specific collection permits were taken for FSD and QTA ecotype from respective institutions, and for GUJ; the plant material was collected with the permission of the field owner. For collecting KK ecotype national guidelines and legislation were followed properly. The Voucher numbers 224-21-01 (KK), 224-21-02 (FSD), 224-21-03 (GUJ) and 224-21-04 (QTA) was provided after identification by senior taxonomist Prof. Dr. Mansoor Hameed from the University of Agriculture, Faisalabad.

### Salinity development in open Field conditions

Two-year field experiment was conducted to evaluate milk thistle's salinity tolerance potential across different ecotypes in terms of yield and biochemical mechanisms, in the New Botanical Garden of the University of Agriculture, Faisalabad. Prior to sowing, ridges were formed to maintain the differences between each soil supplementation, and soil was dug out to a depth of ~ 60 cm to line the trenches with polyethylene sheets, and refilled were lined to avoid the leaching of NaCl. The EC of control and salt stressed plots were 1.2 and 12 dS m^−1^, respectively. It was an attempt to delineate the effect of applied salinity stress (120 mM NaCl) was applied through one time irrigation after selecting sub-optimal lethal level after screening) on the growth, yield, and biochemical attributes and Sily content by using different soil supplementations, including ascorbic acid (500 mM), thiourea (250 µM), and moringa leaf extract (3% MLE), applied with single time irrigation. Plants of four ecotypes, i.e., FSD, GUJ, QTA, and KK were collected from diverse geographical areas and planted under Faisalabad climatic conditions. Seeds of F1 generation were sown under open field conditions for experiments. Seeds were sown at 1–2 cm depth with seeding rate 100 seeds m^−2^. The germination percentage was approximately 70% for all ecotypes, an dplant to plant distance was maintained 60 cm after thinning. The field was divided into two main plots (salinity and control) and with four subplots for each ecotype. Each experimental plot was consisted of 13 lines with each line length of 22.86 m and line to line distance was 60 cm. The two outer lines were acting as guard lines, and the terminal and end of central line’s milk thistle plant was acted as guard plant. Soil supplements were applied after developing salinity stress under Split Plot Design when the plants were at BBCH principal growth stage 3^37^. The plants were harvested when they were at BBCH principal growth stage 5, and a detailed physiological and biochemical analysis was done of shoot and root with three replications per treatment. One set of plants was grown till maturity to collect achenes and determination of their morphological characteristics and Sily contents. The control set of all the ecotypes was kept for elucidating the differences. All the set of plants grown in main- and sub-plots were watered equally with regular intervals to avoid drought stress, when necessary. All chemicals were purchased from Aldrich, Fluka, Merck and Sigma companies, and were of either AR or ACS having high purity grades as per requirement of the performed protocols.

### Growth and yield parameters

Plants were carefully harvested and thoroughly washed with tap water. Shoot length of these plants were measured. Then the plants shoot cut from the roots, and main panicle length (MPL) (cm), and short peduncle length (SPL) (cm) were also measured manually by using routine procedures with three replications per treatment. Half of the fresh plant material was immediately shifted to an ice bath, transported to the laboratory, and preserved at − 40 °C in refrigerator to measure different biochemical analyses^38^.

### Metrological data

The meteorological data were taken from the Agricultural Meteorology Cell, Department of Crop Physiology, University of Agriculture Faisalabad. All values of mean relative humidity, rainfall, and temperature values difference between 2 years during milk thistle growth cycle is given below (Table 1). Soil physico-chemical were also represented in Table 2.

**Table 1:** Weather data during the field trial of 2017 and 2018 at the experimental site.

Year	Temperature (°C)			Relative humidity (%)	Rainfall (mm)	Year	Temperature (°C)			Relative humidity (%)	Rainfall (mm)
2017–2018	Max	Min	Average			2018–2019	Max	Min	Average		
Nov	24.1	11.8	18	84.6	1.5	Nov	27	12.4	19.7	74.6	0.6
Dec	22	6.7	14.4	69.3	4.2	Dec	21.7	6.5	14.1	81.5	0.7
Jan	17.6	8.2	12.9	72	11.5	Jan	21.5	5.5	13.5	75.9	0
Feb	23.3	10.2	16.8	53	4.1	Feb	24	9.5	16.7	73.3	9.5
March	27.3	14.2	20.7	49.5	16.2	March	31.2	16.4	23.8	61.4	12.5
April	37.7	20.9	29.3	30.6	28.3	April	36.8	20.8	28.8	47.3	7.9
Average	25.33	12	18.68	59.83	10.69	Average	27.03	11.85	19.43	69	5.2

**Table 2:** Physio-chemical analysis of the soil of experimental site.

Sample	AB-DTPA extractable		(%)		pH	ECe (dS m^−1^)	mmol L^−1^			SAR
	(mg kg^−1^)									
	P	K	OM	SP			Na^+^	Cl^−^	Ca + Mg	
Faisalabad	2.24	169	1.15	38.29	7.97	0.56	2.39	2	5.1	1.49
Faisalabad	2.15	164	1.06	38.16	8.05	0.58	2.65	2	5.5	1.6
Gujranwala	1.25	159	1.13	35.21	8.2	0.65	3.68	3	5.7	1.5
Gujranwala	1.04	167	0.85	36.24	8.1	0.75	4.03	3	5.9	1.39
Quetta	0.58	244	0.49	41.21	7.86	2.23	8.3	6	18.4	2.73
Quetta	0.44	212	0.34	40.63	7.81	2.29	8.17	6	18.6	2.68
Kallar Kahar	2.2	157	0.81	34.2	8.3	4.2	6.28	4	10.12	1.85
Kallar Kahar	2.1	195	0.85	36.21	8.4	3.9	6.38	5	11.24	1.67

*OM* organic matter, *SP* soluble phosphate.

### Silymarin extraction and determination

Soxhlet extraction was done by using the prescribed protocol of Saleh, et al.^39^. For this purpose, 10 g seeds were placed in thimble holder, and positioned in Soxhlet apparatus. The extraction was carried out by using 200 mL of 80% methanol (prepared in dH_2_O) in the reservoir over 6 h. The absorbance of extracted solution was recorded at 530 nm by using the method of Rahman, et al.^40^. For this protocol, 80% methanol was used as blank. Calibration curve was constructed by dissolving Sily (1 mg/mL) in methanol. After that, 0.18–0.50 mL of standard Sily solution was pipetted into series of 10 mL volumetric flasks. In each flask, 0.003 M potassium permanganate solution was added. Then, solution was diluted to volume with dH_2_O. After that mixture was immediately transferred to a spectrophotometer and decrease in absorbance was recorded for 20 min at 530 nm.

### Oil determination

The amount of seed oil was measured using a Soxhlet apparatus. Seed samples were extracted by continuous extraction with Diethyl ether using Soxhlet extractor and expressed in kg ha^− 1^. Oil yield was calculated as Xie, et al.^41^Eq. (1).

1 $${\text{Oil yield }}\left( {{\text{kg}}\,\,{\text{h}}{{\text{a}}^{ - 1}}} \right) = {\text{Oil content }}\left( \% \right) \, \times {\text{ Seed yield }}\left( {{\text{kg}}\,{\text{h}}{{\text{a}}^{ - 1}}} \right)$$

### Total soluble phenolics

Phenolic (Phe) content was estimated by using the procedure of Julkunen-Tiitto^42^. A 0.1 g of fresh plant sample was homogenized in 1 mL of 80% acetone, after centrifuge supernatant was collected. For 100 µL of extract 1 mL of H_2_O_2_ was added. Then 0.5 mL of Folin phenol reagent was poured in this mixture and shacked vigorously. After that, 2.5 mL of 20% Na_2_CO_3_ was added, then vortexed the mixture. Kept this mixture for 20 min and took the reading at 750 nm, while 80% acetone was used as blank.

### Flavonoids extraction

Flavo were determined by the method developed by Zhishen et al.^43^. To do this measurement, 0.1 g of fresh plant sample was homogenized in 80% acetone. Then 1 mL of extracted solution and 4 mL of distilled water was poured into the test tubes. After that, waited for 5 min, and 0.6 mL of 5% NaNO_2_ and 0.5 mL 10% AlCl_3_ were added to each test tube. After one minute, 2 mL of IM NaOH was added. Reaction mixture was diluted with 2.4 mL of distilled water. Absorbance was taken at 510 nm. For blank 80% acetone was used. The concentration of ascorbic acid content in each sample was calculated from a standard curve plotted with known concentration of ascorbic acid. The total Flavo content of plant was measured by following Eq. (2):

2 $$Y = 0.0205X - 0.1494; \, r = 0.9992$$

where Y is the absorbance, and X is the Flavo content in μg g^−1^.

### Histological studies

For histological studies, the milk thistle ecotype was harvested and preserved to process the tissue for infiltration, cutting sections using microtome as described by Ruzin^44^. Milk thistle seeds was sampled from each ecotype and from same position carefully that immediately was fixed in formaldehyde, acetic acid, alcohol, and water (FAA; 10:5:1:4) for 48 h. The seeds were then transferred to 70% alcohol for long-term fixation until the tissue was processed by microtomy for analyzing P.epi, P.Par and SI thickness. The compound light microscope was used for histological studies.

### Statistical analysis

Main and interactive effects of salinity stress, ecotypes and soil supplementation in milk thistle under field conditions was evaluated on various response variables by analysis of variance (ANOVA) technique at 5% probability level. LSD test was performed to compare the relative means. Standard deviations will be reported where required. Furthermore, Pearson’s correlations were drawn among all the response variables. The lettering on graphs showed significant differences, while no lettering represents non-significant results.

## Results

### Main panicle length

**Figure 1:**
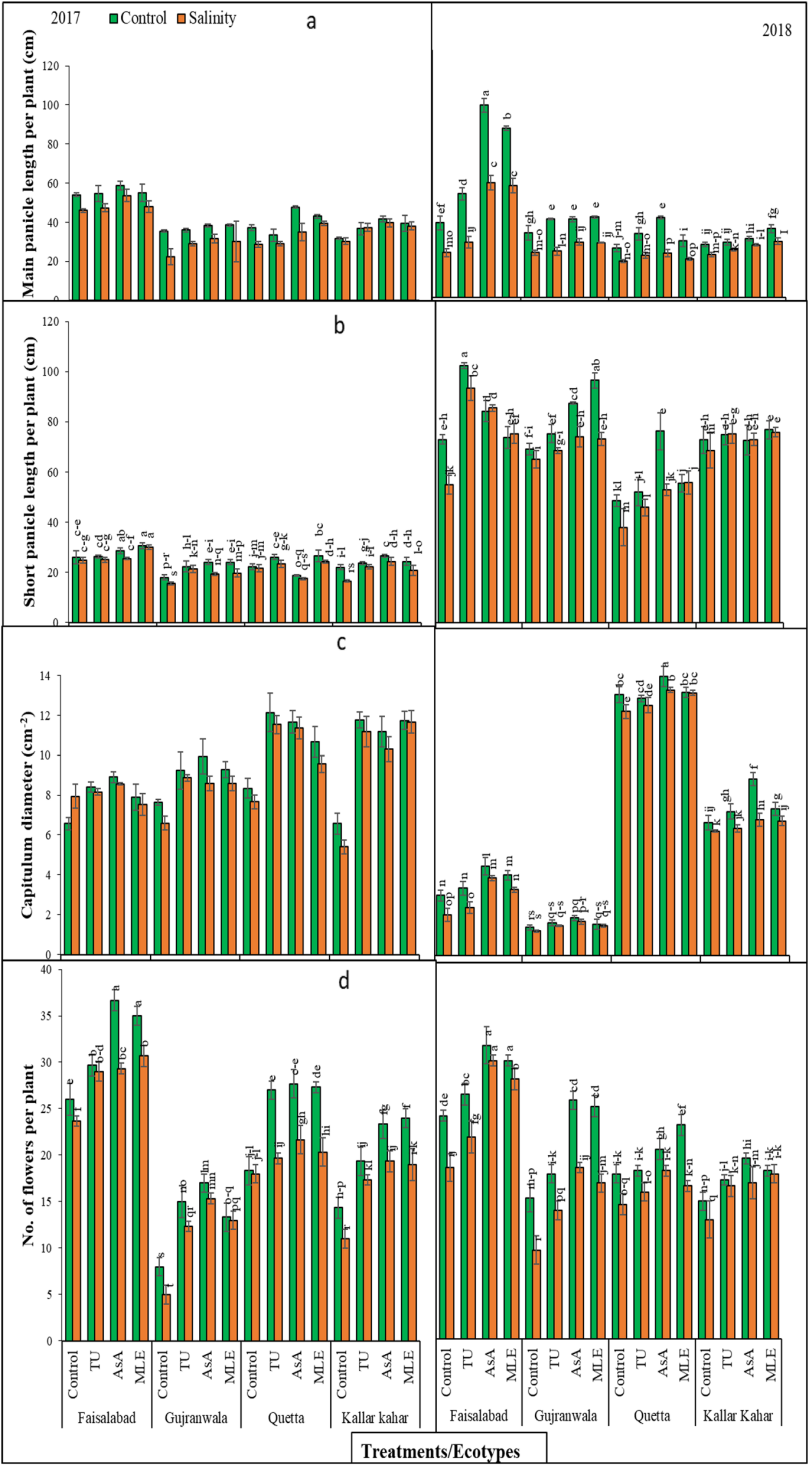
(**a**) Main panicle length, (**b**) short panicle length per plant, (**c**) capitulum diameter, and (**d**) number of flowers per plant of milk thistle under control and salinity stress conditions. The plants were soil supplemented with different plant growth promoters during 2017 and 2018.

Results obtained for MPL showed non-significant (*P* > 0.05) differences in ecotypes and salinity under different soil supplementations, and the interaction among them was also non-significant in 2017. Nevertheless, during 2018 the interaction among salinity, ecotypes, and treatments was significant (*P* < 0.01). During 2017, FSD ecotype displayed substantially increased MPL as compared to other ecotypes under control and stress conditions. Furthermore, exogenous application of AsA was more effective in increasing this attribute in all ecotypes as compared to other PGPs (Fig. 1a).

Data recorded during 2018 revealed that the order of improvement in MPL was: AsA > MLE > TU > Control and AsA > TU > MLE > Control in FSD and QTA ecotypes, respectively, while in GUJ and KK substantial increase was observed in MLE treated plants followed by AsA, TU, and Control showed pronounced results. However, under salt stress condition, this order was obtained higher in AsA supplemented plants followed by MLE, TU and Control in FSD and GUJ ecotypes, while in QTA and KK this order was TU > AsA > MLE > Control and MLE > AsA > TU > Control respectively. On the other hand, highest MPL was recorded in FSD followed by QTA, GUJ, and KK under saline and non-saline conditions.

### Short peduncle length

SPL was significantly (*P* < 0.01) higher in all ecotypes and salinity stress treatments with different PGPs, and the interaction among them was also highly significant in both years (2017–2018). Graphical representation of this attribute showed interesting results when the SPL during 2017 was compared with 2018, a higher SPL was measured in second experimental year while the lowest at the first year of study. However, salinity stress showed a remarkable reduction in SPL during both years.

Under control conditions during 2017, MLE supplementation was better for improving SPL in FSD, GUJ, and QTA ecotypes, while in KK ecotype AsA supplementation showed remarkable results. Data further exposed that under salinity stress, MLE supplementation was quite effective in increasing SPL in FSD and QTA ecotype, whereas in GUJ and KK ecotypes, the effect of AsA was more profound as compared to other supplemented agents (Fig. 1b).

Data noted during 2018 exposed that TU, MLE, and AsA supplementation helped to achieve maximum height for FSD, GUJ, and QTA ecotypes, respectively. Similarly, MLE exogenous supplementation in KK ecotypes gave profound results as compared to other PGPs treatments. Conversely, under salt stress condition TU treatment was better in respect to this attribute in FSD ecotype, while in other ecotypes MLE supplementation was effective in increasing SPL.

### Capitulum diameter

Statistical results for capitulum diameter (CD) exposed significant (*P* < 0.01) differences in ecotypes and salinity under different soil supplementations and the interaction among them was also significant in 2018. However, during 2017 the interaction among salinity, ecotypes, and treatments was non-significant (*P* > 0.05). Under control conditions during 2017 the order of improvement of FSD and GUJ ecotypes was: AsA > TU > MLE > Control and in QTA ecotype the order was effectiveness of increase in CD was: TU > AsA > MLE > Control, while in KK this order was: TU > MLE > AsA > Control. Data further revealed that under salinity stress, the order of changes in this attribute was found as AsA > Control > TU > MLE in FSD, and in GUJ and QTA ecotypes the CD increasing order was: TU > AsA > MLE > Control, while in KK it was noted as TU > MLE > AsA > Control. The order of CD in respect to ecotypes was QTA > KK > GUJ > FSD during 2017 (Fig. 1c).

Considering the results noted during 2018 revealed that soil supplementation of AsA was effective for increasing the CD in all ecotypes under control and stress conditions. On the other hand, highest CD was recorded in QTA followed by KK, FSD, and GUJ under stress and control conditions.

### Number of flowers

Results obtained for number of flowers (NOF) manifested significant (*P* < 0.01) differences in ecotypes and salinity under different soil supplementations and the interaction among these factors was also highly significant for both years (2017–2018).

Under control conditions during 2017 the order of improvement of FSD ecotype was: AsA = MLE > TU > Control: in GUJ ecotype the effectiveness of increase in NOF was: AsA > TU > MLE > Control, while in QTA and KK ecotypes this order was: AsA = MLE > TU > Control and MLE > AsA > TU > Control, respectively. Data further revealed that under salinity stress, the order of changes in this attribute was same as noted in control plants in all ecotypes. On the other hand, highest NOF was counted in FSD followed by QTA, KK, and GUJ under control and stress conditions (Fig. 1d).

Data recorded during 2018 revealed that AsA exogenous application increased the NOF in FSD, GUJ, and KK ecotypes efficiently as compare to other supplied agents, while in QTA ecotype MLE performance was better in enhancing this attribute. Similar results were obtained under salt stress. However, highest NOF was recorded in FSD followed GUJ, QTA, and KK under saline and non-saline conditions.

### Number of achenes per capitulum

**Figure 2:**
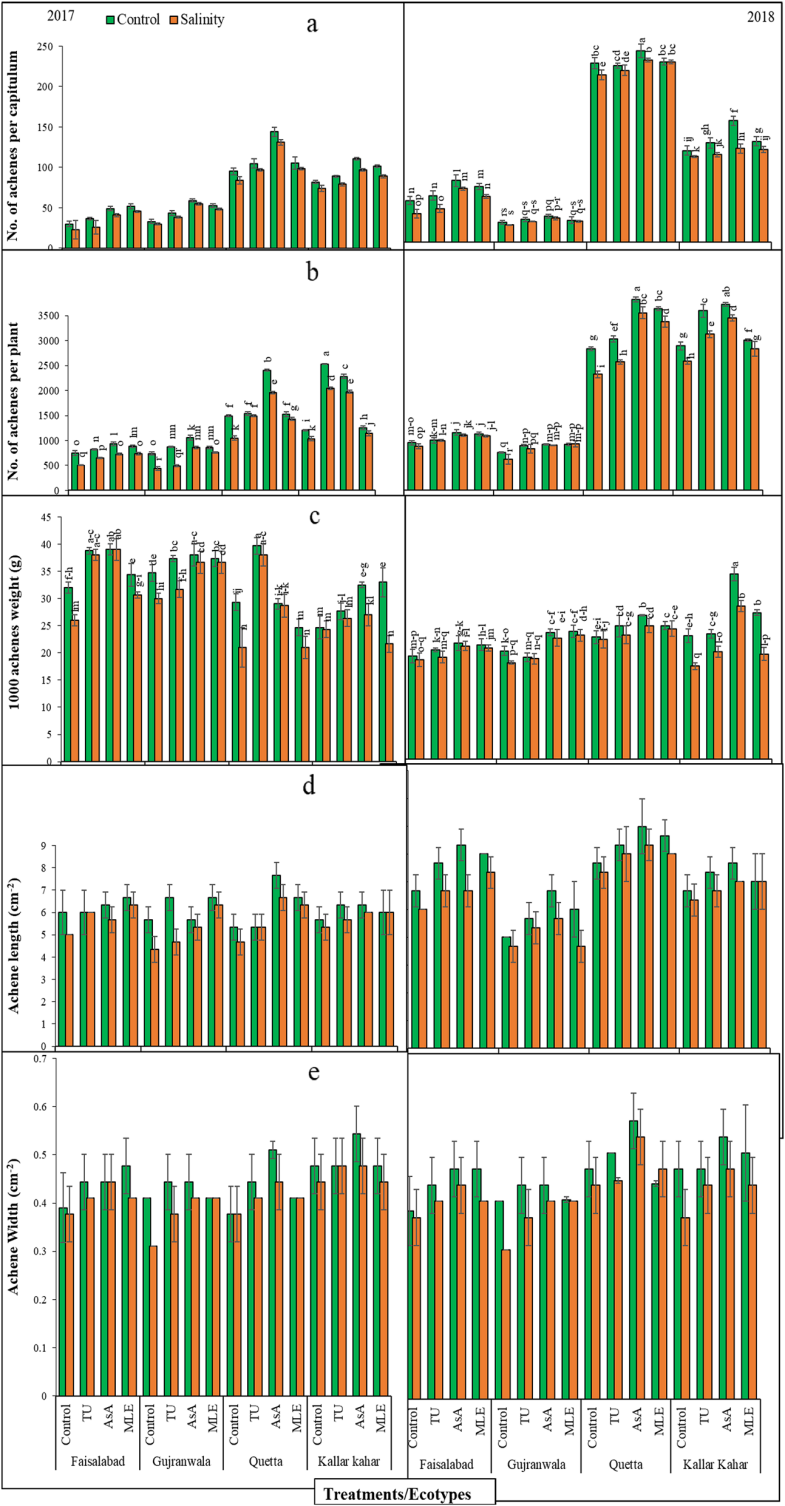
(**a**) No. of achene per capitulum, (**b**) no. of achene per plant, (**c**) 1000 achene weight (**d**) achene length, and (**e**) achene width of milk thistle under control and salinity stress conditions. The plants were soil supplemented with different plant growth promoters during 2017 and 2018.

Results obtained for number of achenes per capitulum (A.C) showed non-significant (*P* > 0.05) differences in ecotypes and salinity under different soil supplementations, and the interaction among them was non-significant in 2017. Nevertheless, during 2018 the interaction among salinity, ecotypes, and treatments was significant (*P* < 0.01). Under control and salt stress conditions during 2017 the order of improvement of FSD ecotype was: MLE > AsA > TU > Control: in GUJ, QTA, and KK ecotypes the effectiveness of an increase in A.C was: AsA > MLE > TU > Control (Fig. 2a).

Nonetheless, during 2018 the order of improvement in control and stress condition was: AsA > MLE > TU > Control in all ecotypes, expect QTA and order was AsA > MLE = Control > TU. Furthermore, A.C was maximum in QTA under saline and non-saline conditions, while minimum was noted in GUJ. The A.C increased with the soil supplementation of all PGPs in different ecotypes, and salt stress caused severe reduction in both year studies.

### Achene yield per plant

Statistical analysis for achene yield per plant (A.P) displayed significant (*P* < 0.01) differences in ecotypes and salinity under different soil supplementations and the interaction among these factors was also highly significant in both years (2017–2018). During 2017 all the soil supplied agents (PGPs) improved this attribute although maximally with AsA in FSD, GUJ and QTA ecotypes, while the order of increase was observed maximum in KK with application of TU. Furthermore, maximum A.P was found in KK, while minimum in FSD control and stress conditions (Fig. 2b).

A comparison of soil supplementation recorded during 2018 revealed that AsA supplementation both under control and salt stress condition was effective in increasing seed yield in all ecotypes. Overall, A.P increased with the soil supplementation of all the treatments in different ecotypes, and salinity stress caused antagonistic effects in both year studies. In contrast, maximum A.P was recorded in QTA followed by KK, FSD, and GUJ under saline and non-saline conditions.

### 1000 achene weight

Results obtained for 1000 achene weight (TAW) manifested significant (*P* < 0.01) differences in ecotypes and salinity under different soil supplementations and the interaction among them was also highly significant in both years (2017–2018). Under control conditions during 2017 the order of improvement of FSD and GUJ ecotypes were: AsA > TU > MLE > Control and AsA > TU = MLE > Control, respectively, in QTA ecotype the effectiveness of increase in 1000 grain weight was: TU > Control > AsA > MLE, while in KK this order was: MLE > AsA > TU = Control. Data further revealed that under salinity stress, the order of changes in this attribute was same as in control for FSD, GUJ, and QTA ecotypes, while in KK it was noted as AsA > TU > Control > MLE (Fig. 2c).

Data recorded during 2018 revealed that AsA efficiently improved the grain weight as compared to other PGPs both under stress and control conditions. Overall, TAW increased with the soil supplementation of all the treatments in different ecotypes in comparison to non-supplemented plants. On the other hand, highest TAW was recorded in KK followed by QTA, GUJ, and FSD under saline and non-saline conditions. Furthermore, salinity stress caused antagonistic effects and reduced grain weight in both year studies.

### Achene length

Results revealed non-significant (*P* > 0.05) difference for achene length (AL) in all ecotypes with different soil supplementations under salinity stress, though the interaction among them was also non-significant (*P* > 0.05) in both years (2017–2018). Under control and salinity stress conditions during 2017, the soil supplementation of MLE attained maximum AL in FSD and GUJ, however, in QTA and KK; AsA was most effective. On the other hand, maximum AL was recorded in QTA followed by KK, FSD, and GUJ under control and stress conditions (Fig. 2d). Similarly, during 2018 the effect of AsA was more profound in increasing AL in all ecotypes under control and salt stress conditions. On the other hand, maximum AL was recorded in QTA followed by FSD, KK, and GUJ under saline and non-saline conditions.

### Achene width

Statistical data demonstrated non-significant (*P* > 0.05) difference for achene width (AW) in all ecotypes with different soil supplementations under salinity stress, however, the interaction among them was also non-significant (*P* > 0.05) in both years of experiment. Under control and salinity stress conditions during 2017 the soil supplementation of AsA attained maximum AW in GUJ, QTA, and KK ecotypes, while in FSD the response of MLE was better as compared to other supplemented agents in control while AsA was better under salinity stress. On the other hand, maximum AW was recorded in KK followed by FSD, QTA, and GUJ under control and stress conditions (Fig. 2e). Similarly, during 2018 the effect of AsA was more profound in increasing AW in all ecotypes under control and salt stress conditions. On the other hand, maximum AW was recorded in QTA followed by KK, FSD, and GUJ under saline and non-saline conditions.

### Total soluble phenolics content

**Figure 3:**
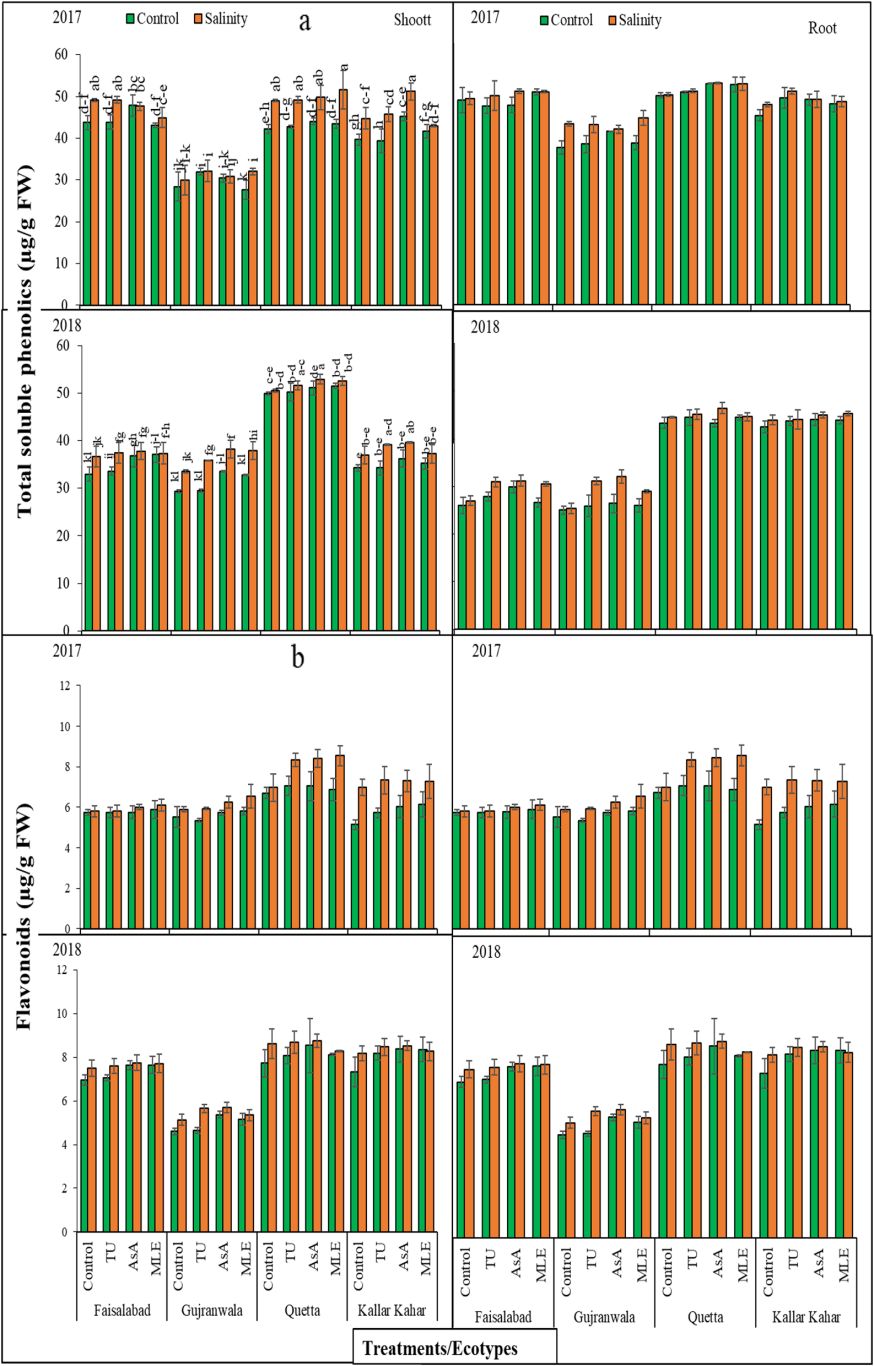
(**a**) Total soluble phenolics content, and (**b**) flavonoid content of root and shoot of milk thistle under control and salinity stress conditions. The plants were soil supplemented with different plant growth promoters during 2017 and 2018.

Results obtained for shoot Phe content manifested significant (*P* < 0.01) differences in ecotypes and salinity under different soil supplementations and the interaction among them was also highly significant in both years (2017–2018). Furthermore, root shoot Phe content was found statistically non-significant (*P* > 0.05) during 2017 and 2018. In 2017, data revealed that the soil supplementation of AsA was more effective in increasing shoot Phe content in FSD, and KK, while in GUJ and QTA, TU supplementation was more effective under control conditions. Under salinity data further explored that TU treatment was effective for FSD and GUJ ecotypes, and MLE treatment showed more improvement in this attribute in QTA ecotype. On the other hand, KK ecotype had more Phe with the soil supplementation of AsA. In 2018, under control condition, AsA was greatly effective in altering this attribute in FSD, GUJ, and KK ecotypes, while MLE supplementation contributed distinctive results in QTA ecotype as compared to other PGPs under control and stress conditions except QTA where under stress AsA was more effective. Ecotypic differences displayed higher Phe content in QTA ecotype in 2017 and 2018 irrespective of the other treatments (Fig. 3a).

Considering root Phe contents of control plants, it was observed that during 2017 QTA ecotype showed higher Phe content followed by FSD, KK, and GUJ under saline and non-saline conditions. While during 2018, QTA = KK depicts maximum root Phe contents followed by FSD and GUJ ecotypes. Furthermore, it was observed that salinity during both experiment years tend to increase the Phe content in all ecotypes in comparison to their control plants. In 2017, under control condition MLE was greatly effective in altering this attribute in FSD and QTA ecotypes while AsA and TU supplementation contributed distinctive results in GUJ and KK ecotypes, respectively, as compared to other PGPs under control and stress conditions. While under stress MLE was effective for FSD and QTA ecotype, while AsA and TU showed more effectiveness for GUJ and KK ecotypes, respectively. Data recorded during 2018 revealed that ASA supplementation increased Phe content in root under stress and control conditions in all ecotypes.

### Flavonoid content

Statistical analysis for shoot and root, Flavo content displayed non-significant (*P* > 0.05) differences in ecotypes and salinity under different soil supplementations, and the interaction among these three factors was also non-significant (*P* > 0.05) in 2017 and 2018. Considering shoot Flavo content of control and stressed plants, it was observed that during both experimental years (i.e., 2017 and 2018) ecotype QTA had higher Flavo content followed by KK > FSD > GUJ under control and stressed plants. During 2017 under control conditions, it was noted that MLE supplementation was effective for all ecotypes except QTA, in which the effect of AsA was at maximum as compared to other PGPs, while under stress the effect of MLE was more effective for all ecotypes. During 2018, under control condition MLE was greatly effective in altering this attribute in FSD and KK ecotypes while AsA supplementation contributed distinctive results in GUJ and QTA ecotypes as compared to other PGPs. Data further explored that, AsA supplementation was effective for all ecotypes under salinity except FSD, in which the effect of MLE was at maximum as compared to other PGPs (Fig. 3b).

Data recorded for root Flavo content revealed that under control condition MLE was greatly effective in altering this attribute in FSD and KK ecotypes while AsA supplementation contributed distinctive results in GUJ and QTA ecotypes as compared to other PGPs, while under stress the effect of MLE was more effective for all ecotypes during 2017. In 2018, a similar trend of increase was obtained as observed during 2017 under control conditions. While under stress the effect of MLE was more effective for all ecotypes. On the other hand, highest root Flavo content was recorded in QTA followed by KK, FSD, and KK under saline and non-saline conditions during 2017 and 2018.

### Silymarin content

**Figure 4:**
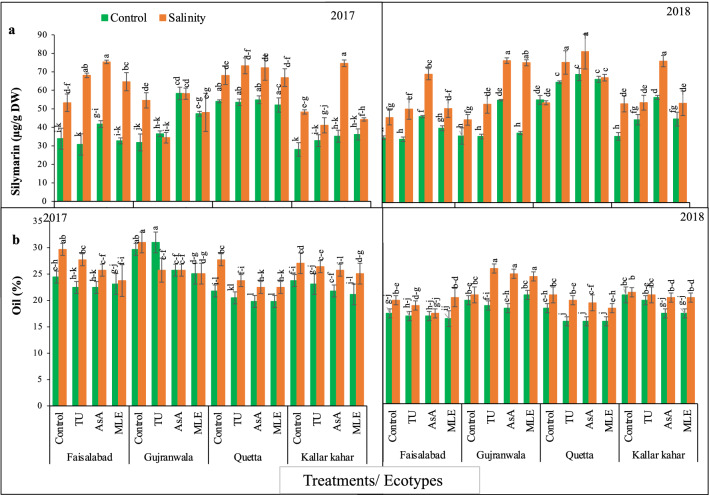
(**a**) Silymarin content, and (**b**) oil content achene of milk thistle under control and salinity stress conditions. The plants were soil supplemented with different plant growth promoters during 2017 and 2018.

Results obtained for Sily content manifested significant (*P* < 0.01) differences in ecotypes and salinity under different soil supplementations and the interaction among them was also highly significant in both years (2017–2018). Under control conditions during 2017 the order of improvement of FSD ecotype was: AsA > MLE = Control > TU: in GUJ ecotype the effectiveness of increase in Sily content was: AsA > MLE > TU > Control, while in QTA and KK this order was: Control = AsA > TU = MLE and MLE = AsA > TU > Control. Data further revealed that under salinity stress, the order of changes in this attribute was found as AsA > TU > MLE > Control in FSD, however, in GUJ, QTA, and KK ecotypes, it was noted as AsA > Control > MLE > TU, AsA = TU > Control > MLE, and AsA > Control > MLE > TU, respectively. On the other hand, highest Sily was recorded in QTA followed by FSD, KK, and GUJ under stress conditions (Fig. 4a).

Data recorded during 2018 revealed that the order of improvement in FSD, GUJ, QTA, and KK under control condition was: AsA > MLE = Control > TU, AsA = MLE = TU = Control, AsA = MLE = TU > Control and AsA > MLE = TU > Control, respectively. However, under salt stress this order was: AsA > MLE > TU > Control in FSD ecotype, AsA > MLE > TU > Control in GUJ, AsA > TU = MLE > Control in QTA and AsA > MLE = TU = Control in KK.

### Oil content

Results obtained for oil content showed significant (*P* < 0.01) differences in ecotypes and salinity under different soil supplementations and the interaction among them was also highly significant during 2017 and 2018. Data recorded for oil content revealed that all soil supplementation treatments were highly effective in lowering oil content irrespective of stress and ecotypes differences during 2017. On the other hand, highest oil content was recorded in GUJ followed by FSD, KK, and QTA under saline and non-saline conditions during 2017 (Fig. 4b). Considering oil content of control plants, during 2018 the trend of increase in this attribute was note as: Control > TU = AsA > MLE in FSD ecotype, and in GUJ ecotype the effectiveness was: MLE > Control > TU > AsA, while in QTA this order was: Control > TU = MLE = AsA, and in KK the trend was found as: Control > TU > MLE = AsA. Data recorded under salinity stress, in FSD this order was: MLE > Control > TU > AsA, and in GUJ ecotype the effectiveness was: TU = AsA = MLE > Control, while in QTA this order was: Control > TU > AsA > MLE, and in KK the trend was found as: Control = TU > AsA = MLE.

### Anatomy

**Figure 5:**
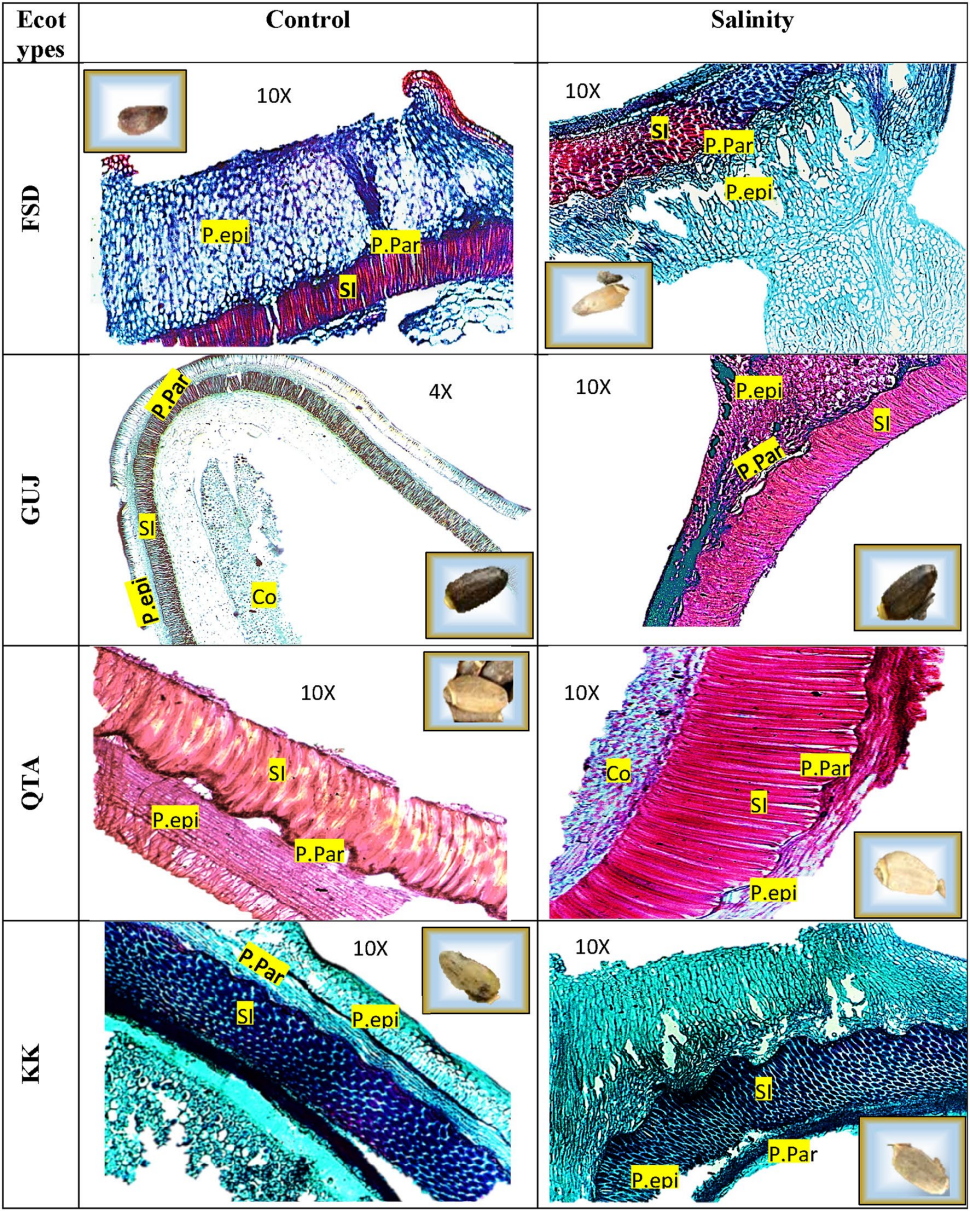
Elucidation of salinity stress treatment on the achene color and achene P.par and P.epi and SI length in different milk thistle ecotypes. Co, cotyledon; SI, seed integument; P-Par, pericarp parenchyma; P-epi pericarp epidermis.

Seed color variations were observed in all the ecotypes under control and stress conditions. The seed coloration was shiny, smooth and flat achene. The ecotypic color varies from dark brown for GUJ, brown for FSD and brown for QTA and KK. The epidermis of the P.epi is made up of a single layer of cells that are normally radially elongated and only occasionally folded or collapsed; it is quite thick and of a cellulose-pectic nature and originated from the outermost tangential walls. P-par were multiple layers of parenchymal cells (7–10). SI, an epidermis shaped by radially elongated and tightly packed macro-sclereids with external macro-sclereids consists of the seed integument. Statistical data revealed significant (*P* < 0.01) differences in salinity and ecotypes with respect to P-epi, P-Par and SI. Interestingly, P.epi, P.Par and SI thickness were higher under saline conditions as compared to control conditions in all ecotypes and the trend of improvement was QTA > KK > FSD > GUJ. The pericarp thickness was more profound during 2017 as compared to 2018, due to more harsh weather conditions (Fig. 5; Table 3).

**Table 3:** Depiction of mean values of pericarp epidermis (P-epi), pericarp parenchymal cell layer (P-Par) and seed integument epidermis (SI) as influenced under control and salinity stress during 2017 and 2018.

Ecotypes	Conditions	P-epi (μm)		P-Par (μm)		SI (μm)	
		2017	2018	2017	2018	2017	2018
Faisalabad	Control	19C	21C	19F	9G	35D	39D
	Salinity	21C	29A	42C	52C	39C	45C
Gujranwala	Control	16D	20C	14G	14F	35D	35E
	Salinity	27AB	22C	34D	34D	37CD	35E
Quetta	Control	26B	27AB	30E	13F	57B	57B
	Salinity	29A	29A	65A	58B	68A	69A
Kallar Kahar	Control	25B	25B	20F	18E	39C	28F
	Salinity	26B	26B	58B	65A	60B	67A
LSD * P* ≤ 0.05		E = 0.00; S = 0.00; E × S = 0.00 E = 0.00; S = 0.00; E × S = 0.00		E = 0.00; S = 0.00; E × S = 0.00 E = 0.00; S = 0.00; E × S = 0.00		E = 0.00; S = 0.00; E × S = 0.00 E = 0.00; S = 0.00; E × S = 0.00	

Different letters within the same column indicate statistically significant differences, critical value for comparison (CVC).

CVCP-epi 2017: E = 1.88; S = 1.32; E × S = 2.65: CVCP-epi 2018: E = 1.67; S = 1.18; E × S = 2.37.

CVCP-Par 2017: E = 2.57; S = 1.82; E × S = 3.64: CVCP-Par 2018: E = 2.28; S = 1.61; E × S = 3.23.

CVCSI 2017: E = 2.22; S = 1.57; E × S = 3.14: CVCSI 2018: E = 2.66; S = 1.88; E × S = 3.76.

### Correlation

During 2017, the pearson’s correlation data showed strong linear correlation of SI with P.Par, P.epi and Sily, irrespective of salinity treatments.

**Figure 6:**
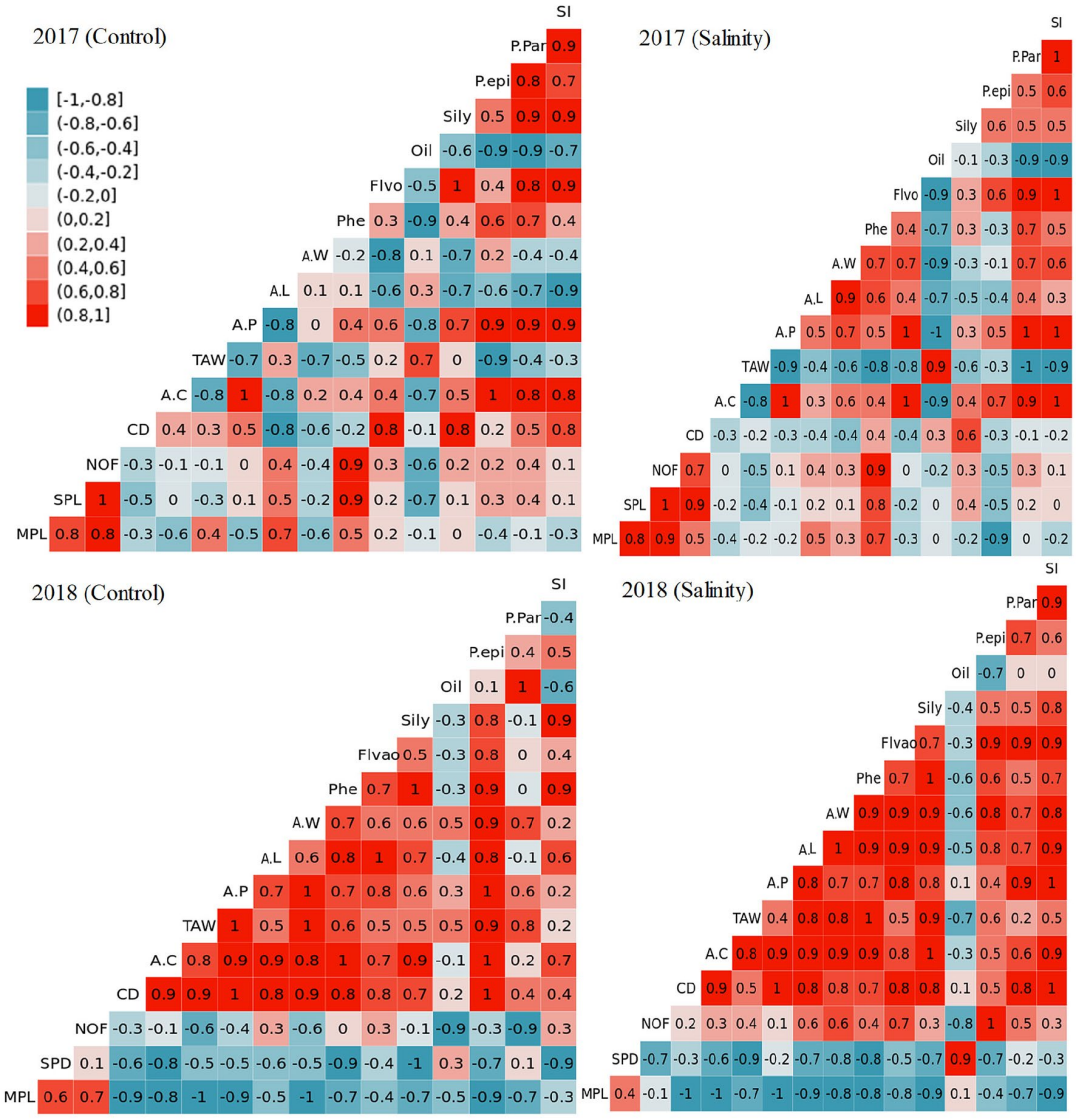
Pearson’s correlation matrix between different growth and yield parameters under control and salt stress conditions during 2017 and 2018. Correlation matrix of different dependent variables with independent variables (ecotypes) under control stress conditions during 2017. *SI* seed integument epidermis, *P-Par* pericarp parenchyma, *P-epi* pericarp epidermis, *Sily* silymarin, *Flavo* flavonoids, *Phe* Phenolics, *AW* achene width, *AL* achene length, *A.P* achene/plant, *TAW* thousand achene weight, *A.C* achene/capitulum, *CD* capitulum diameter, *NOF* number of flowers, *SPL* short peduncle length, *MPL* main panicle length. Blue box represents negative correlation, while red for positive correlation.

Oil content showed negative correlation with P.Par, P.epi and Sily and SI under control and stress conditions. Flvo and Phe had positive correlation with P.Par, P.epi and Sily, while negative with oil contents. AW showed strong negative correlation with P.epi and Sily, SI, Flavo and Phe, and positive with oil and P.epi under control conditions, but under stress conditions strong linear correlation was seen with Flvo and Phe, P.par and SI while negative with Sily, oil and P-epi. AL was positively related with AW, Phe, oil, and negative correlated with Flvo, P.Par, P.epi and Sily and SI under control conditions, while under stress AL had strong linear association among AW, Phe, Flvo, P.Par and SI, and negative with oil, Sily and P-epi. Besides, A.P had negative relation with AL and oil, and positive with AW, Phe, Flvo, P.Par, P.epi, SI and Sily under control conditions, while under salinity stress it showed strong linear association with AW, Phe, Flvo, P.Par, P.epi, SI, AL and Sily, and negative with oil contents. TAW was positively associated with AL, oil and Flvo, and negatively linked with A.P, AW, Phe, P.Par, P.epi and SI, while no relation was seen with Sily contents under control conditions. Under salt stress, TAW was negatively associated with AL, Flvo, A.P, AW, Phe, P.Par, P.epi and SI, and positively correlated with oil. In addition, A.C was negatively associated with TAW, AL and oil, while positively linked with Flvo, A.P, AW, Phe, P.Par, Sily, P.epi and SI under control and stress conditions. Under control conditions, CD was positively associated with A.C, TAW, oil, Flvo, A.P, P.Par, P.epi and SI, and negatively correlated with AL, AW and Phe, while under stress it showed negative correlation with A.C, TAW, Flvo, A.P, P.Par, P.epi, AL, AW and SI, and positive association with Sily, oil, and Phe. NOF showed negative correlation with CD, A.C, TAW, AW, and strong linear correlation with AL, Phe, Flvo, Sily, oil, P.Par, P.epi and SI, and no relation with A.P under control conditions. NOF was strongly related with CD, AW, and linear correlation with AL, Phe, A.P, Sily, P.Par and SI, and negatively correlated with A.C, TAW, A.P, Flvo, oil and P-epi. Moreover, SPL positively associated with NOF, AL, Phe, Flvo, sily, P.epi, P.par and SI, while negatively linked with CD, A.C, TAW, AW, and oil, and no relation was seen with A.C under control conditions. SPL showed negatively relation with Flvo, sily, P.epi, A.C, TAW, and oil, while positively linked with NOF, CD, AL, AW, Phe, Sily, P.Par and SI under stress. MPL was positively correlated with SPL, NOF, TAW, AL, Phe and Flvo negatively related with CD, A.C, A.P, AW, oil, P.epi, P.par and Si, and no relation with sily contents under control, while under stress it showed linear relation with SPL, NOF, CD, AL, AW, Phe while negatively related with A.C TAW, A.P, Flvo, Sily, P.epi and SI, and no relationship was seen while observing oil and P.Par (Fig. 6).

During 2018, P.par showed a linear relationship with SI under control while negative under salt-stressed conditions. Moreover, P.par showed a positive correlation with P.par and SI under both stress and control conditions. Under control conditions, oil content was strongly associated with P.par as compared to P.epi, while a negative correlation was seen with SI. In addition, oil content under salt stress conditions showed negative relation with P.epi, and no relation was seen while considering P.par and SI. Sily content showed a negative correlation with oil and P.par, and positive association with P.epi and SI under control conditions, while under salt stress it was negatively associated with oil, and positively correlated with P.epi, P.par and SI. Flvo and Phe contents were negatively associated with oil while positively linked with sily, P.epi, P.par, and SI under control and stress conditions. AW showed a positive correlation with Flvo, Phe, sily, P.epi, P.par and SI under control conditions, while a negative correlation was the seed with oil contents under stress. AL showed a strong linear relationship with AW, Flvo, Phe, Sily, P.epi, and SI, and negatively associated with oil contents and P.par under un-stressed conditions, while under salt stress conditions negative association was seen with oil content only. Under both control and stressful conditions, A.P was positively associated with AL, AW, Flvo, Phe, sily, P.epi, SI, oil, and P.par. TAW showed a positive correlation with A.P, AL, AW, Flvo, Phe, sily, P.epi, SI, oil, and P.par under control conditions, while a negative correlation was the seed with oil contents under stress. A.C was positively associated with TAW, with A.P, AL, AW, Flvo, Phe, sily, P.epi and SI, and negative with oil and P.par under control conditions, while under stress negative correlation was observed with oil contents only. Furthermore, CD was positively linked with A.C, TAW, A.P, AL, AW, Flvo, Phe, Sily, P.epi, SI, oil, and P.par irrespective to salinity treatment. NOF showed a negative correlation with CD A.C, TAW, A.P, AL, AW, Sily, P.epi and P.par, and positive with AL, Flvo and SI, and no relationship with Phe under control conditions, while under stress negative relation was seen only for oil contents. Additionally, SPL showed a negative link with CD A.C, TAW, A.P, AL, AW, Sily, P.epi, AL, Flvo, and SI, and positive with P.par, oil and NOF under control conditions. However under salt stress positive relation was only seen with oil contents. MPL showed a negative relationship with CD, A.C, TAW, A.P, AL, AW, sily, P.epi, AL, Flvo and SI, P.par and oil, while positive with SPL and NOF, and under stress conditions positive relation was seen with SPL and oil (Fig. 6).

## Discussion

Milk thistle has gained considerable importance due to the presence of Sily in the achene pericarp, which is used for the treatment of hepatitis patients^45^. In Pakistan, limited work has been considered out especially carrying on Sily contents of milk thistle. Pakistan has been blessed with a variety of agro-ecological zones, where the behavior and growth performance of some species differs considerably. So, keeping in view this question presumably milk thistle ecotypes were collected from four ecological niches of Pakistan in order to determine their salinity tolerance potential as well as the role of medium supplementation of different known PGPs on the growth yield of milk thistle ecotypes.

It has been reported that milk thistle shows the variable response in salinity from different world ecological zones^19,20,46^, and salinity response ranges from 9 to 30 dS/m. In the current study, 12 dS m^−1^ NaCl (sodium chloride) was applied to four milk thistle ecotypes; collected from FSD, GUJ, QTA and KK, all the ecotypes were medium supplemented with predetermined level of PGPs such as TU (250 µM), AsA (500 µM) and MLE (3%), and the data was recorded after four months. The ecotype from QTA was the most salt-tolerant under control as well as salinity stress for responsiveness to medium supplementation of various PGPs. Among the PGPs the best response was recorded with soil supplementation of AsA for MPL and SPL (Fig. 1).

The overall performance of any plant is assessed in terms of ultimate yield which is outcome of number of integrated parameters. The floral shape and development are important key parameters in this regard^47^. It has been observed that among the different reproductive growth and yield parameters such as NOF, CD number of A.P, TAW, and achene size the QTA ecotype followed by KK surpassed the rest of the ecotypes (FSD and GUJ) both under control and stress conditions (Figs. 1 and 2). It was further accessed from the data that soil supplementation of AsA was the most effective followed by MLE as compared to TU and treatment set; where no supplementation was made. The improved reproductive growth and yield parameters were resulting from soil supplementation in all ecotypes not only under control but also under salt stress conditions. This revealed that soil supplementation of these agents is not only important in improving growth parameters but also their effects of carried out the latest stages (reproductive growth and developmental stages) of milk thistle which is an important outcome of this study.

Salinity is an adverse environmental abiotic factor for plant growth and development as known for resting the cell elongation and expansion, thereby causing a reduction in overall plant growth and development. The difference in the ecotypes for salinity and soil supplementation can be attributed to their inherent ability to withstand this adverse factor. It is important to mention that no previous study from Pakistan has identified milk thistle ecotypes, which may witness the milk thistle response to salt stress. In this study, all growth and yield attributes were significantly affected by salinity stress. Our findings were inconsistent with Ghavami and Ramin^20^ studies, who reported that growth and yield components reduced with salinity greater than 9 dS m^−1^ in all genotypes. The correlation data for different growth and yield parameters revealed that there was a difference between two years among the behavior of ecotypes for salt response.

Plants subjected to grow under stress conditions tend to accumulate the secondary metabolites in higher quantities as compared to their non-stressed counter parts^48^. This indicates a clear shift in the metabolic pathways leading to the expenditure of more energy in the operation of secondary metabolites. Keeping in view the importance of secondary metabolites in stress tolerance, the changes in the concentration of Phe, Flavo, oils and Sily (a flavonolignan) were measured (Figs. 3, 4). Data showed that a variety of Phe were predominately accumulated in the shoot as compared to root, while soil supplementation of the PGPs was helpful in considerably increasing their concentration, further leading to the confirmation of anticipated role in salinity tolerance. The PGPs effectively detoxify the adversities of salinity stress, and improved overall growth related attributes, which may leads to higher Sily production in its achene part.

The concentration of all these secondary metabolites was relatively higher during 2018, while ecotypes obtained from QTA excitably others in showing greater content of all these secondary metabolites. Among the soil supplementation, AsA followed by MLE was more helpful in promoting the production of all these secondary metabolites. This may be due to the reason that AsA has been reported to play a positive role in modulating the metabolic activities in the cell, while MLE which contains a mixture of various growth enhancers also appeared to promote the cell metabolic activities^49^.

A thorough survey of literature has revealed that Phe has been accumulated in a variety of plants in various parts under both abiotic and biotic stress, and there exists a positive association in the accumulation of Phe and stress tolerance of various species. Likewise, alkaloids and saponins are also known to play a role in the abiotic stress tolerance such as salt, drought and heat, although studies are limited but the most important bound in this study is the flavonolignan popularly known as Sily for which the milk thistle is known, has entirely scantly investigated for changes in its levels under abiotic stress such as salinity and soil supplementation of various PGPs. It is important to notice that Sily being increasingly reported for its medico-pharmaceutical role in treating the liver, kidney, and diabetic diseases^3^. So in the present study, it was noticed that salt stress enhanced the accumulation of Sily in all ecotypes but QTA and KK ecotypes were leading in accumulating this important secondary metabolite (Fig. 3). Likewise, soil supplementation of AsA and MLE further promoted its contents in the salinity stressed plants. While formulating the hypothesis it was conceived that since adverse growth factors induced the accumulation of secondary metabolites, the levels of Sily may also be enhanced during 2018 (high temperature, low rain fall) and salinity stressed conditions. The change was proven after the analysis of Sily in the achenes of milk thistle. This showed that applied salinity had modulated the Flavo biosynthesis pathway in the achene probably at latter stages of its maturation, and all the soil supplementations played a profound role in the Sily accumulation in the pericarp. However, a possible way of Sily accumulation in the achenes is not certain yet^50^.

The milk thsitle oil extraction from its seeds is beneficial for human health. These seeds have a relatively high oil content (26–31%) comparable with most oilseeds^51^. In fact, the oil is a by-product of Sily production, and its seeds contain a relatively high amount of oil (20–25%)^52–54^. In present study oil content was higher in GUJ ecotype followed by FSD, KK, and QTA ecotype (Fig. 4). Correlation data exposed that milk thistle oil content had negative correlation with TAW, A.P, AL, AW, Phe, Flavo and Sily, but showed no correlation with NOF, AC and MPL, while positive correlation was seen with SPL. Our findings showed consistency with findings of Fick, et al.^55^. According to them oil content of inbred lines of milk was negatively correlated with achene weight, but showed no correlation with flowering, plant height and head diameter.

In addition, the fruit morpho-anatomy revealed significant variations among the ecotypes mainly under saline stressed conditions. The P.epi, P.par and SI length varied significantly with the stress exposure and seed color density, which shows a comprehensive histo-localization of Sily. Milk thistle SI is rich source of Sily and its precursor taxifolin^56^. On the contrary, in the present study strong correlation was seen between SI, P.epi and P.Par thickness with Sily content, which is also confirmed with strong linear correlation of Sily with achene size (AL and AW). Seed color variations were seen in all ecotypes both under salt stress and control conditions. FSD and GUJ seeds were brown and dark brown in color, respectively, while in QTA and KK the fruit was hard skimmed achene which is shiny, smooth, flat, and light brown in color. Giuliani, et al.^56^ also observed seed color variations among different genotypes, and reported that dark color of fully ripened achene is due to the accumulation of tannins in pericarp subepidermal layer, which probably results from impairment of Flavo biosynthesis. According to Hewitt and Ford^57^ condensed tannins also impaired the seed quality of grains. So, the Sily content varied with ecotypic color variations higher in QTA and KK, while lower in GUJ and FSD, more in salinity stress as shown in Fig. 5. The here described color variation could represent a favorable characteristic for the possible employment of milk thistle achenes for Sily extraction as by-products in the economic sector. In the year 2017, there was no relationship among most of the parameters of respective growth and yield, while presence of positive relationship of most of the growth and yield attributes with Sily and seed pericarp (P.epi, P.par and SI) representing relatively adverse environmental conditions prevailing the year 2018. So, we believed that Sily which is a pharmaceutically and economically important secondary metabolite obtained from milk thistle can be enhanced by growing this plant under mild to moderate levels of salinity, and seed with light color can be selected for more Sily extraction.

## Conclusion

Salinity registered an adverse effect on growth and yield of all the ecotypes, while the soil supplementation especially of AsA and MLE emerged more effective in overcoming the adverse effect of ionic toxicity. This was evident from improved growth and yield attributes. The metabolic response of ecotypes from QTAand GUJ was quite distinct with regard to salinity tolerance and soil supplementations. Salinity recorded an adverse effect on growth and yield of all the ecotypes, while the soil supplementation especially of AsA and MLE emerged more effective in overcoming the adverse effect of ionic toxicity. Soil supplementation of different PGPs effectively improved Sily and oil contents by improving growth and yield related attributes, especially under salinity stress. In this work, the morpho-anatomical structure of Milk thistle achene showed significant variations under salt stress with achene color with respect to ecotypic variations especially under stress conditions. It is recommended that light brown achene ecotypes such as QTA and KKmay be tailored fast for extracting more silymarin and fulfilling the needs of pharmaceutical industry under saline areas. Further studies may be conducted to enhance the silymarin biosynthesis using modern biotechnological and biochemical techniques.

## Data Availability

The data that support the outcomes of current experimentation are available from the corresponding author upon reasonable request. We confirm that all the experimental research and field studies on plants (either cultivated or wild), including the collection of plant material, complied with relevant institutional, national, and international guidelines and legislation.

## Abbreviations

FSD: Faisalabad
GUJ: Gujranwala
QTA: Quetta
KK: Kallar kahar
AsA: Ascorbic acid
TU: Thiourea
MLE: Moringa leaf extract
SI: Seed integument
P-Par: Pericarp parenchyma
P-epi: Pericarp epidermis
Sily: Silymarin
Flavo: Flavonoids
Phe: Phenolics
AW: Achene width
AL: Achene length
A.P: Achene/plant
TAW: Thousand achene weight
A.C: Achene/capitulum
CD: Capitulum diameter
NOF: Number of flowers
SPL: Short peduncle length
MPL: Main panicle length
PGPs: Plant growth promoters
